# Reversal flow in the left anterior descending artery after internal thoracic artery grafting

**DOI:** 10.1186/s13019-022-02008-0

**Published:** 2022-10-10

**Authors:** Hiroyuki Nakajima, Akitoshi Takazawa, Yoshitsugu Nakamura, Hatsue Ishibashi-Ueda, Akihiro Yoshitake, Atsushi Iguchi

**Affiliations:** 1 Department of Cardiovascular Surgery, International Medical Center, Saitama Medical University, 1397-1 Yamane Hidaka, Saitama, 350-1298 Japan; 2 Department of Pathology, National Cerebral and National Cardiovascular Center, Suita, Osaka Japan

**Keywords:** Off-pump coronary artery bypass graft, Internal thoracic artery, Intimal hyperplasia, Hypoperfusion syndrome

## Abstract

**Background:**

The flow capacity of the in situ internal thoracic artery (ITA) is not necessarily sufficient and can be a cause of hypoperfusion syndrome. We present a catastrophic case of in situ ITA grafting for an isolated left main trunk obstruction 13 years after the modified Bentall operation.

**Case presentation:**

A 33-years-old woman had undergone the modified Bentall operation. Coronary angiography showed a critical stenosis in the left coronary artery. The patient underwent emergency off-pump coronary artery bypass graft with the left ITA to the left anterior descending artery (LAD). On the 7th day, the patient had severe dyspnoea and hypotension. Catheter angiography showed that the ITA was patent; however, blood flow from the in situ ITA was delayed, and reversal flow from the apex to the proximal LAD was found. The patient underwent implantation of a left ventricular assist device.

**Conclusions:**

Concomitant aortocoronary bypass to the circumflex branch will minimise the risk of hypoperfusion, especially for young patients without atherosclerotic disease.

**Supplementary Information:**

The online version contains supplementary material available at 10.1186/s13019-022-02008-0.

## Background

Hypoperfusion syndrome is defined as myocardial ischaemia occurring despite a fully patent lumen of a coronary artery bypass graft (CABG). It typically occurs in the operating room or intensive care unit. We present a case of such a lethal sequela that occurred 1 week after CABG.

## Case presentation

**Fig. 1:**
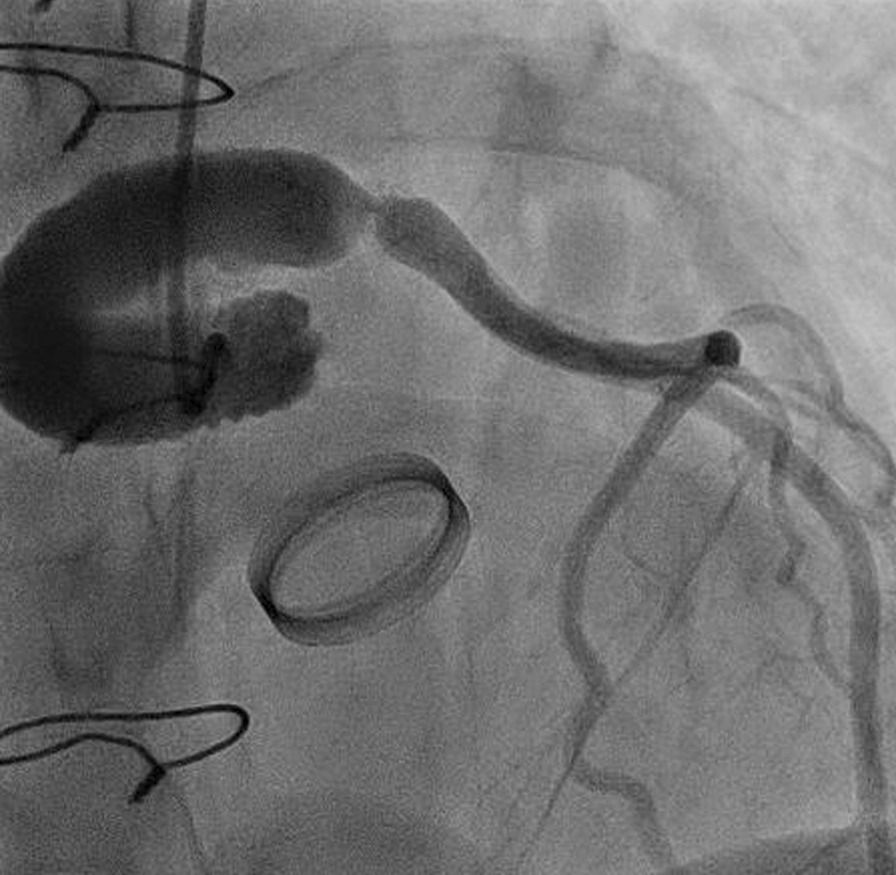
Modified Bentall procedure with artificial graft interposition between the orifice of the left main coronary artery and the aortic root was performed. A critical stenosis occurred at the anastomotic site

A 33-years-old woman with Marfan syndrome had undergone a modified Bentall operation at the age of 20 years. The patient recently experienced progressive chest pain on exertion. Coronary angiography showed a critical stenosis in the left main trunk, which was anastomosed using a 10 mm diameter artificial vascular graft (Fig. 1). The right coronary artery, which had been directly anastomosed with the aortic root, was intact. The patient was diagnosed with unstable angina. Considering her young age and unusual stenotic lesion, CABG was preferred over percutaneous coronary intervention.

The patient underwent emergency off-pump CABG with the left internal thoracic artery (ITA) to the left anterior descending artery (LAD) through a left anterior mini-thoracotomy because of the risk of repeat median sternotomy and the possibility of future aortic surgery in the setting of Marfan syndrome. On the second postoperative day, the patient was extubated. The international normalised ratio (INR) of prothrombin time was 1.42. Then, intravenous heparin and oral aspirin were started. Activated partial thromboplastin time (APTT) was controlled more than 1.5 times the normal value, which was 45 s, until INR exceeded more than 2.0. However, the patient complained of chest discomfort and dyspnoea. Catheter angiography was performed and showed that the ITA graft was patent with a normal flow to the entire LAD (Additional file 1: Video 1).

**Fig. 2:**
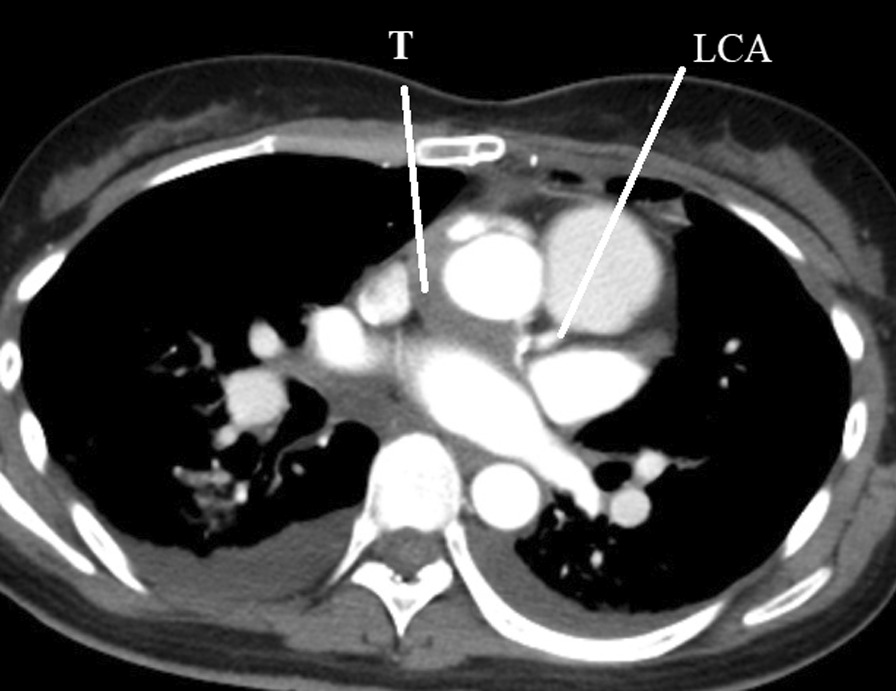
Contrast-enhanced computed tomography revealed that the interposing artificial graft to the left coronary artery was occluded by a thrombus. T, thrombosis in the interposing graft; LCA, left coronary artery

On the 7th day, platelet count was 180,000; INR was 1.71; and APTT was 48 s with the administration of heparin, warfarin and aspirin. However, contrast-enhanced computed tomography revealed that the interposing artificial graft to the left coronary artery was occluded by thrombosis (Fig. 2). On the same day, the patient had severe dyspnoea and hypotension during rehabilitation. Emergency catheter angiography showed that the ITA was patent; however, blood flow from the in situ ITA was delayed, and reversal flow from the apex to the proximal LAD and left circumflex coronary artery was found (Additional file 2: Video 2). Intra-aortic balloon pumping and cardiopulmonary circulatory support were immediately started, and an additional saphenous vein bypass graft was created between the left subclavian artery to the left circumflex artery through a left anterior thoracotomy.

On the 11th postoperative day, the patient was transferred to our hospital, and a left ventricular assist device was implanted. The patient was considered a candidate for heart transplantation. However, she died of intracranial haemorrhage 4 months after initial ITA grafting.

**Fig. 3:**
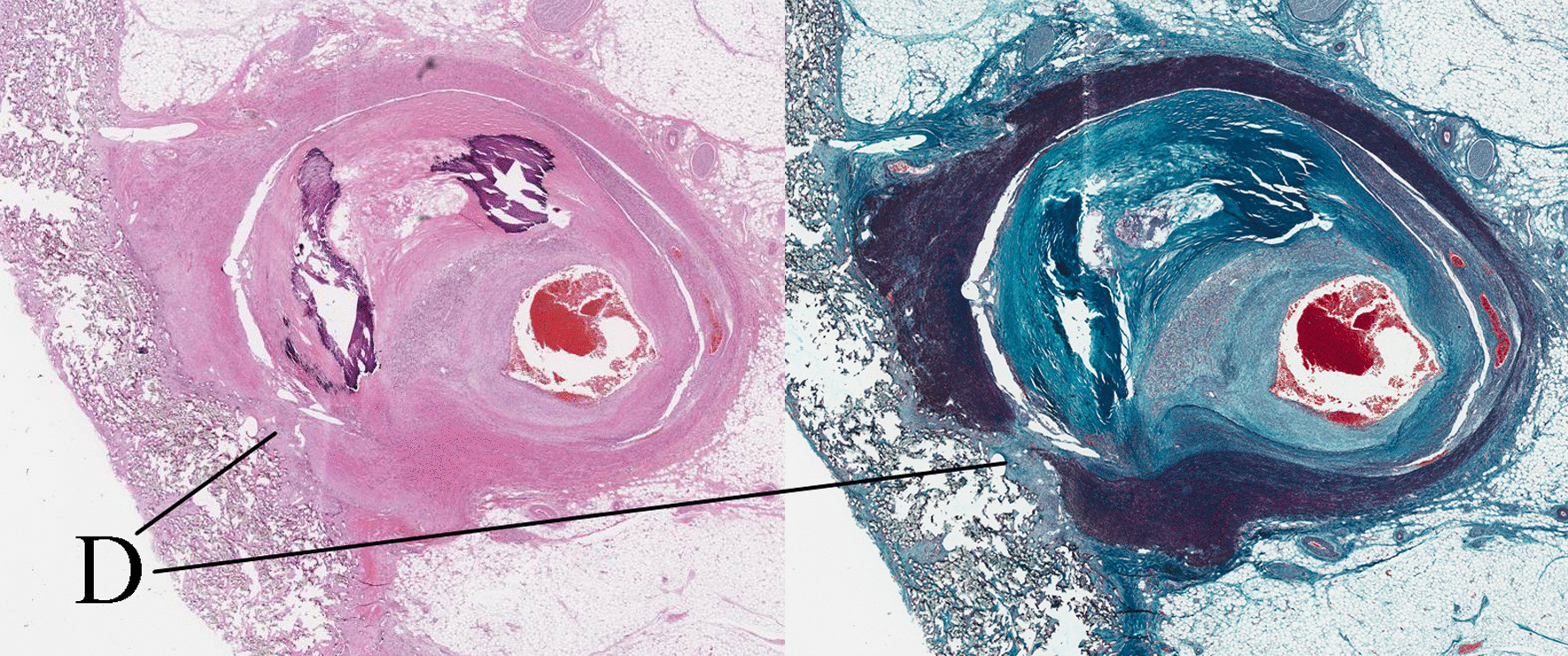
(Left) Haematoxylin–eosin stain. (Right) Masson’s trichrome stain. Stenosis of the left coronary artery was caused by intimal hyperplasia, which was associated with a defect of adventitia (D)

Autopsy revealed a broad subendocardial myocardial infarction. The cause of the severe ostial stenosis of the left coronary artery was intimal hyperplasia, which was associated with an adventitia defect (Fig. 3) [1].

## Discussion and conclusions

Loop et al. [2] have reported that a principal mechanism of hypoperfusion syndrome was graft–recipient artery mismatch. Specifically, hypertrophic heart, small ITA graft, 90% stenosis of the large LAD and normal ventricular contraction were reported to be common features [3]. These features were manifested by the patient in this case report. Additional bypass grafting with a saphenous vein graft has been recommended for such a harmful situation [3, 4]. In patients with chronic atherosclerotic disease, chronic exposure to low perfusion pressure induces collateral vasculature and hibernation of the myocardium, probably causing the myocardial tissue to become adaptable to lower perfusion pressure, and a single ITA could be reliably used to re-vascularise a large area, such as three-vessel coronary regions [5].

In the patient in this case report, the clinical course should not be considered a technical or strategical error, but unpredictable negative results. The ITA graft was functioning normally at the beginning of the postoperative course. However, decreased antegrade flow in the artificial graft due to ITA grafting and relatively large diameter of the interposing graft resulted in thrombosis. The possibility of underlying coagulopathy cannot be denied because the coagulation factors in a patient receiving warfarin therapy for mechanical valve could not be precisely examined. The created bypass grafts could not attain blood flow as the sole blood source of the left coronary artery. Therefore, peripheral vascular resistance could not be adapted to attain the required flow from the subclavian artery.

In conclusion, concomitant aortocoronary bypass to the circumflex branch is recommended, especially for young patients who have left main trunk stenosis without chronic atherosclerotic disease, even if a minimally invasive surgery seems attractive in some aspects.

## Supplementary Information

**Additional file1**. **Video 1**: On the second postoperative day, catheter angiography showed that the internal thoracic artery graft was patent with a normal flow to the entire left anterior descending artery.

**Additional file2**. **Video 2**: On the 7th postoperative day, blood flow into the left anterior descending artery (LAD) was obviously worse than that 5 days earlier. The apical portion of the LAD was filled by collaterals from the right coronary artery. Blood flow into the LAD was reversed.

## Data Availability

Datasets used or analysed during this study are available from the corresponding author on reasonable request.

## Abbreviations

APTT: Activated partial thromboplastin time
CABG: Coronary artery bypass graft
INR: International normalised ratio
ITA: Internal thoracic artery
LAD: Left anterior descending artery
